# Urgency enforces stimulus-driven action across spatial and numerical cognitive control tasks

**DOI:** 10.1371/journal.pone.0322482

**Published:** 2025-05-02

**Authors:** Anika Krause, Christian H. Poth

**Affiliations:** 1 Differential Psychology, Personality Psychology and Psychological Assessment, Department of Psychology, Bielefeld University, Bielefeld, Germany,; 2 Neuro-Cognitive Psychology, Department of Psychology, Bielefeld University, Bielefeld, Germany; University of Bologna, ITALY

## Abstract

It has been shown that urgency in cognitive control tasks elicits a time-window in which responses are dominated by stimuli rather than goals. If stimulus information conflicts with goal-relevant information, urgency impairs goal-directed responses. This was shown for an antisaccade task as well as tasks using manual responses. Critically, however, all previous studies on manual responses used arrows as stimuli, leaving it unclear whether urgency affects cognitive control also in tasks using different stimuli. Here, we show that the urgency effect can also be elicited in three cognitive control tasks that do not use arrow stimuli. Participants completed either a Spatial Stroop task with word stimuli, in which they reacted to the word meaning while ignoring the spatial position, or a Numerical Stroop Task, in which they had to respond to the numerically larger of two presented numbers. The physical size of the numbers varied but was irrelevant to the task. The third task was a Simon task, where participants were instructed to react to the color of a stimulus while ignoring its spatial position. In all tasks, urgency evoked a time window, in which the position or the physical size dominated the response, which was evident from a drop of performance below chance level in conflict situations. These results reveal that the effect of urgency on cognitive control does not depend on arrow stimuli and emerges in a number of other spatially related tasks, specifically in spatial and numerical cognitive control tasks. As such, they suggest that urgency affects cognitive control more generally.

## Introduction

Cognitive control enables humans to act according to goals and intentions by allowing to execute goal-directed actions even when conflicting action tendencies are triggered by stimulus information [1–4]. Recently, it has been shown that urgency (time-pressure) opens up a time-window, in which response are dominated by stimulus driven-information and cognitive control is overpowered. This could be shown for saccadic eye movements [5] as well as for manual cognitive control tasks [6]. However, previous studies that investigated the effect of urgency on cognitive control in manual tasks always used arrows as stimuli [6,7], which are simple and overlearned stimuli automatically associated with spatial directions [8]. In this study, we were able to show that the effect of urgency on cognitive control also occurs in tasks that use different types of stimuli. Thus, urgency seems to enforce stimulus-driven action across cognitive control tasks.

Goal-directed behavior requires that information from the environment is coordinated with internal information like current goals and intentions [1,9]. Cognitive control plays a crucial role in this process. Cognitive control allows to maintain goal-directed action, while stimulus information from the environment triggers conflicting actions [1–3]. In cognitive conflicts, two mechanisms work against each other. One reaction is automatically evoked by the stimulus and is rather involuntary and impulsive (bottom-up control). The other reaction is relevant to the goal and must be voluntarily selected and executed (top-down control). The impulsive and non-task-relevant reaction must be suppressed, so that the goal-directed reaction can be executed. This type of impulse control is attributed to cognitive control [4]. Thus, cognitive control provides the basis for executing goal-directed action.

Recently, it has been shown that, when urgency in a cognitive control task limits the time to respond to a stimulus, irrelevant but salient stimulus information dominates the response in a given time window. In such situations, a correction of this triggered response by cognitive control is no longer possible. This was first shown for saccadic eye movements [5] and subsequently for manual responses [6]. In an antisaccade task, in which participants are normally instructed to look away from a suddenly appearing stimulus [10–12], although such a stimulus naturally captures the gaze [13,14], Salinas et al. [5] limited the time available for responding to this stimulus. In high-urgency situations, contrary to the instruction, the gaze was almost always captured by the sudden onset of the stimulus. The performance dropped below chance level within a certain time window, because a cognitively controlled correction of the naturally (visually) evoked response was no longer possible. In the study of Poth [6] this paradigm was adapted and transferred to a spatial cognitive control task (the Spatial Stroop task, in which the spatial position of the stimulus conflicts the meaning of the stimulus) and a cognitive control task, in which the cognitive conflict is not based on the spatial location of the stimulus, but arises between the meaning of the stimulus and the meaning of surrounding distractor stimuli (the Eriksen Flanker task). In these tasks, too, high urgency led to a temporary drop of performance below chance level due to the execution of the stimulus-driven response.

In spatial cognitive control tasks, such as the Spatial Stroop task used by Poth [6], a conflict arises between the position of a stimulus and its meaning. A stimulus can be presented either on the right or on the left side of the screen. The position of the stimulus is irrelevant to the task and the response should be based on the stimulus meaning. Position and meaning can match (congruent condition) or differ (incongruent condition). Incongruence leads to a cognitive conflict that becomes apparent in slower responses and lower accuracy [15]. The spatial component of these tasks drives the cognitive conflict. The strength of such spatial cognitive conflicts can be influenced by spatial attention [7].

Therefore, attention in spatial cognitive control tasks could be organized in priority maps. In a priority map of spatial attention the bottom-up saliency as well as the top-down goal-relevance of a target are integrated [16,17]. Priority maps are topographic maps of space representing all objects in a visual scene. Based on these priority maps, locations in space are selected for further processing and thus ultimately preferred for responding [16–18]. Recently, it has been shown that tying attentional resources to a location in space by giving an additional fixation task, reduces the strength of processing of the spatial location of a stimulus in the periphery and subsequently reduces the strength of the cognitive conflict [7]. This argues for a shared spatial system, in which both stimuli in space and the cognitive conflict in spatial cognitive control tasks are processed. The sudden onset of a stimulus at a certain position is highly salient, thus leading to strong responses in priority maps [19,20], a prioritization of the corresponding location for reaction and ultimately strong behavioral responses in favor of the stimulus location [11,12,14]. A conflict arises between this automatically triggered response and the goal-directed action. Therefore, due to the high salience of the location in spatial cognitive control tasks, in high urgency conflict situations the response to just this location is dominating, whereas the response to the stimulus meaning is not executed.

In the used Eriksen Flanker task with arrow stimuli [21,22], the cognitive conflict is between the direction of the target arrow and the direction of the surrounding flanker arrows. In the center of the screen, the target stimulus is presented. It is presented surrounded by flanker stimuli, which are shown in equal numbers to the right and left of the target stimulus. These flanker stimuli can either match the target’s direction (congruent condition) or deviate from it (incongruent condition) and thus elicit the same or the opposite response. In incongruent trials, a conflict arises between the two responses. The stimuli are always presented in the center of the screen, so that the position does not vary. Thus, in general, the task itself does not involve a spatial conflict, but evokes a stimulus-stimulus conflict between the target stimulus and the flanker stimuli.

However, critically, both tasks in the study of Poth [6] used arrow stimuli. Arrows are highly overlearned symbols that automatically direct one’s visual attention into the direction the arrow points to [8]. Studies showed that reactions that follow the presentation of an arrow stimulus are influenced by the spatial direction the arrow is associated with, even when it is task-irrelevant [23,24]. Thus, arrows seem to be closely related to spatial attention mechanisms. Since the task-relevant stimulus dimension determines the task, it is still unclear, whether the effect of urgency on cognitive control may be specific for arrow stimuli. Therefore, we vary the task-relevant dimension here. In this study, we ask, whether the effect of urgency on cognitive control can also be elicited in tasks that use different types of stimuli and thus include further cognitive processes such as reading, numerical processing and color processing. That is, we investigated the effects of urgency on cognitive control performance in a Spatial Stroop task with letter stimuli [15,25,26], a Numerical Stroop task [27–29] and a Simon task [15,30–32].

In the Spatial Stroop task with word stimuli, the German words for “left” (i.e., “LINKS”) and “right” (i.e. “RECHTS”) are either presented on the left or on the right side of the screen, eliciting a conflict between the position of the word and its’ meaning. The meaning of the word is defined as the goal-relevant stimulus feature while the position is irrelevant to the task [15,25]. In the Numerical Stroop task, two numbers are presented, one on each side of the screen. The numbers vary in their numerical and physical size. Participants are instructed to press the mouse button corresponding to the side on which the numerically larger number is presented while ignoring the numbers physical size. In incongruent situations a conflict arises between the physical and the numerical size of the numbers [27–29]. Both tasks derive from the classic color-word Stroop task, in which a color word written in color is presented, causing a cognitive conflict between the color and the meaning of the word in incongruent trials [33].

In the Simon task, participants have to react to the color of a dot while ignoring its spatial position. Participants are instructed to react to one color with a left mouse button click and to the other one with a right mouse button click. As in the Spatial Stroop task, the dot can be presented either on the left or on the right side of the screen. Thus, the color and the position can either evoke the same response or a different one, eliciting a cognitive conflict [15,30–32]. Both the position as well as the physical size of a stimulus are highly salient features that can trigger automatic responses. This can result in a cognitive conflict between this automatically triggered response by position or physical size and the goal-relevant response. All these tasks have in common that it is not possible for participants to completely ignore the irrelevant feature eliciting a congruency effect with longer reaction times in situations when the salient but irrelevant stimulus feature is incongruent to the target feature compared with trials with correspondence between the triggered responses [15,28].

In order to investigate the effect of urgency on cognitive control in these tasks we used the urgency paradigm proposed by Salinas et al. and Poth [5,6]. Urgency was manipulated by setting the response interval to one second beginning with a Go-Signal and simultaneously varying the duration of a gap between the Go-Signal and the presentation of the target stimulus. Participants are instructed to focus on reacting within the response interval. If the gap between the Go-Signal and the presentation of the target is short, urgency is low, since enough time remains to process the target and execute the response. However, if the gap duration is long, urgency is high. The performance (proportion of correct reactions) will be measured over the raw processing time (rPT), which is defined as the time from presentation of the target to the response. For negative and very short rPTs responses are mainly guesses, since the response had to be initiated before the presentation of the actual target stimulus. For longer rPTs, the responses are driven by the irrelevant as well as the relevant stimulus dimension.

We expected that in the congruent condition, in which the information regarding position and meaning or physical and numerical size trigger the same response, performance should be around chancel level at first and should then increase with declining urgency. In the incongruent condition, in which a cognitive conflict arises, urgency should lead to a time window, in which the reaction is dominated by the goal-adverse stimulus information and thus performance should drop below chance level. After this drop, with decreasing urgency, performance should recover up to chance level and then increase up to near-perfect performance.

## Methods

Experiments 1, 2B and 3 were preregistered before data collection on the Open Science framework (Experiment 1: https://osf.io/cun4t; Experiment 2B: https://osf.io/rf8gm; Experiment 3: https://osf.io/yt8ge). Experiment 2A was preregistered as part of a larger project (https://osf.io/s4w3z).

### Ethics statement

The experiments followed the ethical regulations of the German Psychological Society (DGPs) and were approved by Bielefeld University’s ethics committee. All participants gave written informed consent before participating.

### Participants

All experiments were conceptualized as Small-N-Designs [34,35], in which a small number of participants is investigated with a very large number of trials. Specifically, we collected 5940 trials per person and therefore 29700–35640 trials per experiment. Small-N-Designs use a parametric experimental approach, which means that a large number of interval-scaled levels of the independent variable is acquired per participant in order to assess the dependent variable as a psychometric function of the independent variable. In this study, using this approach, we can study the evolution of performance over the time of the rPT, which is why the approach is established in this research field [5–7]. The sample sizes for these experiments were chosen in advance and are based on previous studies in this research field [5–7] as well as statistical considerations. The experiments were performed by five (Experiment 2A and 2B) and six (Experiment 1 and 3) participants. Participants were recruited and data collected from April 22^nd^, 2022 to November 17^th^, 2023. In Experiment 1, one participant had to be excluded due to incorrect task understanding (namely the participant reacted to the word position instead of the word meaning), so that in Experiments 1, 2A and 2B the data of 5 participants were analyzed (Experiment 1: 3 female and 2 male aged 19 to 32 years, *M* = 24.4, *Md* = 22, *SD* = 5.41; Experiment 2A: 4 female and 1 male, aged 22 to 28 years, *M* = 24.8, *Md* = 24, *SD* = 2.59; Experiment 2B: 4 female and 1 male, aged 22 to 24 years, *M* = 23.2, *Md* = 23, *SD* = 0.84). In Experiment 3, the data of 6 participants were analyzed (3 female and 3 male aged 19 to 27 years, *M* = 23.67, *Md* = 24, *SD* = 2.66) All participants had normal or corrected-to-normal vision.

### Experimental setup

The experiments were performed in a dimly lit room. The participants were placed in a distance of 71 cm (Experiment 1, 2B and 3) or 57 cm (Experiment 2A) from the computer screen. In order to obtain a stable value for the luminance, the screen was preheated before starting the experiment (parameters based on Poth & Horstmann [36]). In Experiment 1, 2B and 3, the head of the person was stabilized by a chin-and-forehead-rest which ensured a standardized distance to the screen. The monitor was a CRT-Monitor of the manufacturer View Sonic (Brea, California, USA; model Graphics Serie G90fB; 36 cm x 27 cm) with a refresh rate of 100 Hz and a resolution of 1024 x 768 pixels. The screen was controlled by a graphics card of the type GeForce GTX 970 (driver version 344.48, NVIDIA, Santa Clara, California, USA). In Experiment 2A, the monitor was a 47 cm x 29 cm sized monitor from Samsung Electronics Company (South Korea; model 2233RZ) with a refresh rate of 100 Hz and a resolution of 1680 x 1050 pixels. The screen was controlled by a graphics card of the type GK104 (NVIDIA, Santa Clara, California, USA).

The experiments were controlled by the Psychtoolbox3 [37,38] and the Eyelink Toolbox (Experiment 1, 2B and 3 [39]) and ran under the use of the program MATLAB in the version R2014b (Experiment 1, 2B and 3) or version R2019a (Experiment 2; The MathWorks, Natick, Massachusetts, USA). In addition, in Experiment 1, 2B and 3, the eye movements of the participants were recorded by a video-based and tower-mounted Eyetracker (EyeLink 1000, SR Research, Ontario, Canada) with a measuring rate of 1000 Hz. The Eyetracker was set with a 9-point grid calibration and recorded the participant’s right eye. Additionally, in all experiments, a wired computer mouse was used for assembling the participants responses.

All stimuli in the experiments were presented in front of a grey background (RGB = 128, 128, 128). As fixation stimulus a black square (0.1° x 0.1° visual angle; RGB = 0, 0, 0; < 1 cd/ m²) was presented in the center of the screen. The feedback stimuli were either a green star and a red exclamation mark (Experiment 1) or a smiling and sad looking smiley (Experiment 2 and 3). The target stimuli differed between the experiments and are described separately below.

#### Experiment 1.

In the first experiment, a Spatial Stroop task with word stimuli was used. The target stimuli were the words “LINKS” and “RECHTS”, German for left and right. The words were written in font Arial in font size 20 (2.2° x 0.9° visual angle for “LINKS”; 3.1° x 0.9° visual angle for “RECHTS”) and in font color black (RGB = 0, 0, 0; < 1 cd/ m), using only capital letters. The stimuli could be presented either on the left or on the right side of the screen on the horizontal axis at a distance of 6° visual angle from the center of the screen. Accordingly, the location and meaning of the word could match (congruent trials) or differ (incongruent trials). The participants’ task was to press the mouse button that matched the meaning of the word, i.e., the right button for the word “RECHTS” and the left button for “LINKS”. The position of the stimulus was irrelevant and should be ignored.

#### Experiment 2.

The second experiment consisted of a Numerical Stroop task. Experiment 2B provides a replication of Experiment 2A. This was done to prevent that our findings reflected a false positive result. In the Numerical Stroop task, two numbers were presented, one on the right and one on the left side of the screen, each at a distance of 4° visual angle (measured from the center of the digit) from the center. The numbers varied in their numerical and physical size. All numbers were presented in black font color (RGB = 0, 0, 0; < 1 cd/ m) and font Arial. We used two physical sizes. In each trial, the physically larger number was presented in font size 76 (1.6° x 3.2° visual angle), the smaller number in font size 40 (0.8° x 1.7° visual angle). Regarding the numerical size, the numbers could differ by 1, 2, or 5 units. The possible number combinations were taken from Dadon & Henik [28] and are shown in Table 1. Subjects were instructed to press the mouse button that corresponded to the position of the numerically larger number. If the numerically larger number was presented on the right, the right mouse button should be pressed and vice versa. The physical size could be congruent to the numerical size, meaning that the numerically larger number was also physically larger, or incongruent, so that the numerically larger number was the physically smaller of the two numbers. The physical size of the numbers was irrelevant to the task and should be ignored.

**Table 1:** The different combination of number stimuli in Experiments 2A and 2B.

Distance	Number stimuli
1	1–2, 3–4, 6–7, 8 - 9
2	1–3, 2–4, 6–8, 7 - 9
5	1–6, 2–7, 3–8, 4 - 9

In addition to this project, the present Experiment 2B was also part of another project investigating the reportability of the subjective relaxation state across multiple experiments. Therefore, the Relaxation State Questionnaire (RSQ [40]) was additionally collected in a pre-post design. However, the results are not relevant to the study reported here and will be reported later as part of the other project (Steghaus & Poth, in prep.).

#### Experiment 3.

The third experiment was a Simon task. The target stimuli were colored dots with a diameter of .42° visual angle that were either green (RGB = 0, 90, 0; 7.30 cd/ m) or blue (RGB = 0, 0, 250; 7.32 cd/ m). The stimuli were presented at a distance of 6° visual angle from the center either on the left or on the right side of the screen. Participants were instructed to ignore the position of the stimulus and to react to the color by pressing the left mouse button if the stimulus was green and the right mouse button if it was blue.

### Procedure

Each of the experiments consisted of 5 sessions of 1188 trials each, for a total of 5940 trials per participant. Within the blocks, the possible combinations of all variables occurred equally often and were presented in randomized order. In Experiments 1 and 3, each session was divided into nine blocks of 132 trials each. Since Experiments 2A and 2B contain more potential stimuli, they consisted of 3 blocks of 396 trials each. Between blocks, subjects had the opportunity to take a short break. In each experiment, 132 practice trials were initially presented.

**Fig 1:**
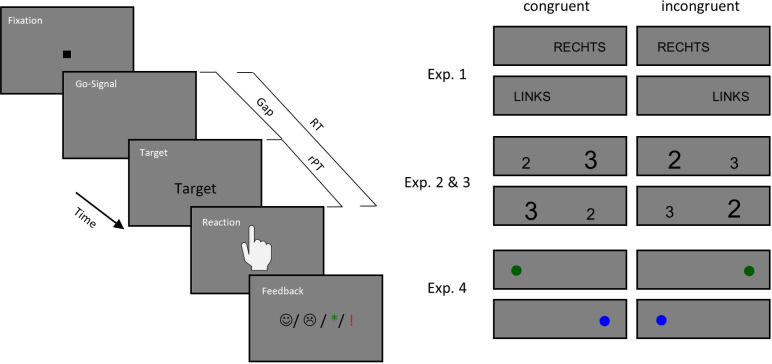
Experimental paradigms. Each trial started with the presentation of the fixation stimulus for 350, 400 or 500 ms. The disappearance of the fixation stimulus was the Go-Signal, which marked the beginning of the response interval of 1000 ms. After a variable gap of 0–950 ms (0, 100 200 … 900, 950 ms) the target appeared. In Experiment 1, the target was either the word RECHTS (German for right) or the word LINKS (German for left) presented on the left or on the right side of the screen. Reactions should match the meaning of the word. In Experiment 2A and 2B in each trials two numbers were presented, one on each side of the screen. The numbers varied in their numerical as well as their physical size. The reaction should match the side of the screen, on which the numerically larger number was presented. In Experiment 3, participants had to react to the color of a dot (green or blue). The spatial position of the dot had to be ignored. After their reaction participants received a feedback regarding the punctuality of their response. If they responded within the predetermined interval they received a positive feedback, a green star or a smiling emoji. For a delayed response the feedback was negative, a red exclamation mark or a sad emoji was presented. The feedback was shown for 750 ms.

The experimental paradigms are shown in Fig 1. Each trial began with the presentation of the fixation stimulus for a period of 350, 400, or 500 ms. The disappearance of the fixation stimulus served as a Go-Signal and marked the beginning of the response interval of one second. Within this second, the response had to be executed. Participants were instructed to react to the stimulus within this response deadline. They should respond in time, even if this means that they have to execute (i.e. guess their response) or at least prepare their response before the stimulus has been presented. The target appeared after a variable gap from 0 to 950 ms in 11 gradations (0, 100, 200, …, 900, 950 ms). The length of the gap reflected the degree of urgency of the trial. The longer the gap, the less time remains from the response interval to perceive the target stimulus, process it, and initiate the response. The target was presented until the response. In trials with long gap durations, participants had to prepare their response before the target was presented in order to react within the response deadline. As suggested by Salinas et al. [5] and Poth [6] it can be assumed that during the gap participants prepare both possible responses, so that both associated motor plans are active simultaneously and compete for execution.

After participants press a mouse button, the feedback stimulus appeared for 750 ms. If the participants managed to respond within the response deadline, they received a positive feedback (star or smiling smiley). If they reacted too late, the negative feedback stimulus (exclamation mark or sad looking smiley) was presented. The feedback referred only to the punctuality of the response, correctness was not considered. Since participants tend to emphasize accuracy and avoid guessing, which happens at the cost of failing to respond within the response deadline, they received this feedback regarding the timing of their response. The feedback helps participants to learn the response deadline as well as it motivates responses that meet the deadline.

### Data analysis

Data and analysis code are available on the Open Science Framework (https://osf.io/knf8w/). Data were analyzed using R (4.1.0, https://www.R-project.org/). The analysis was performed according to the same scheme for all experiments. Since Experiment 2B is a replication of Experiment 2A, we collapsed the data and analyzed a larger dataset with N = 10 participants. As such they are described in the following results section. The individual results of the tachometric analyses are reported in the Supplemental Material (see SM1).

Before the analysis, the practice trials (132 trials) were excluded. In addition, only trials with rPT values in the range from -200 to 1000 ms were considered for the analysis. If not stated otherwise, all analyses refer to this rPT range. Negative rPT values refer to reactions that were not yet target driven, since they were executed before the target was even presented.

Performance in terms of accuracy was defined as the proportion of correct responses and calculated accordingly. In addition, the raw processing time (rPT) was calculated for each trial. The rPT is defined as the difference between the reaction time (RT) and the length of the gap (rPT = RT - gap duration). It represents the time available to perceive and process the target and to initiate the response. In the following analyses, the rPT was considered as an independent variable [5]. For rPTs from -200 to 1000 ms the average proportion of correct answers was calculated for a running bin of 1 ms. Afterwards, the performance was locally regressed on these rPT bins using the loess-function of R with a span parameter of 0.2. The loess-function of R is a nonparametric method for smoothing data, which uses multiple local regressions to fit the performance over the rPT [41]. As the data were concordant across all participants, the data analysis was performed on the aggregated data. The tachometric functions show the evolution of the performance over the time of the rPT. This type of analysis allows to evaluate the contribution of the task-relevant information and the task-irrelevant information to the response with a high temporal resolution, which is why this type of analysis is established in this field of research [5–7]. For each experiment, two tachometric functions were plotted, one for each of the congruency conditions. The minima of the curves were compared using a permutation test, which is a robust non-parametric statistical method. The permutation test locates the original difference of the minima between the congruent and the incongruent condition in a distribution of effects from a 1000-fold reanalysis of the raw data with a randomized labelling of the congruency condition. As effect size for the permutation tests Cohen’s *d*_*z*_ was calculated as the empirical difference of the minima divided by the standard deviation of the permutation distribution

Furthermore, we performed a repeated measures ANOVA as alternative analysis method, as suggested by one reviewer. Therefore, we divided the rPT in bins of 100 ms for a range from 0 to 1000 ms and selected the bins in which a value for the performance was given for each person and each congruency condition. Then we compared the performance between the congruency conditions and the rPT bins using a repeated measures ANOVA. Sphericity was tested using Mauchly’s test for sphericity. If necessary, we applied the Greenhouse-Geisser sphericity correction. Afterwards, post-hoc paired t tests with Bonferroni correction were performed. Cohen’s *d* was calculated as effect size. To investigate the interaction between the congruency and the rPT bins, the congruency effect was computed as the difference in performance between congruent and incongruent trials and compared over the rPT bins. Afterwards, post-hoc paired t tests with Bonferroni correction were performed. As effect size we calculated Cohen’s *d.*

## Results

### Experiment 1: Spatial Stroop task

**Fig 2:**
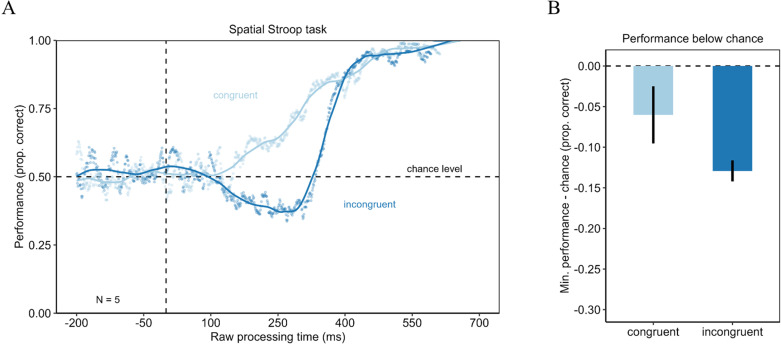
Urgency evokes dominance of stimulus-driven information in a Spatial Stroop task with words. Left panel shows the tachometric functions for the Spatial Stroop task with word stimuli in the congruent and incongruent condition. In the congruent condition performance started around chance level and then increased with increasing rPT. In the incongruent condition, however, the performance started at chance level but then dropped below chance level before the performance recovered and increased with increasing rPT. Right panel shows the difference of the minimum of the tachometric functions minus chance level for the congruent and the incongruent condition. The incongruent condition evoked a larger drop below chance level. Error bars are standard errors from a bootstrap.

The tachometric function, a psychometric function representing the accuracy as a function of the rPT, revealed a qualitative difference between the congruency conditions. The performance in the congruent condition was initially around chance. From an rPT of around 100 ms onwards, performance increased to a near-perfect performance. The tachometric function in the incongruent condition showed a qualitatively different course. While performance also started around chance level, it then dropped clearly below chance level for an rPT around 250 ms. After this drop, performance also increased to near-perfect performance (see Fig 2A). To test for a significantly deeper drop in performance in the incongruent condition (see Fig 2B), the minima in performance were compared between the congruent (minimum = .44) and the incongruent condition (minimum = .37) using a permutation test. The test revealed a significant difference with *p* < .001 (*d*_*z*_ = 4.87). Urgency seems to enable stimulus-driven information to overcome goal-directed information in a specific time-window.

A repeated measures ANOVA was computed for rPT bins from 0 ms to 700 ms. The ANOVA revealed a significant main effect for the rPT bins (*F*(5, 20) = 21.41 with *p* < .05 with Greenhouse-Geisser correction; η ^2^_G_ = .71) as well as for the congruency condition (*F*(1, 4) = 52.98 with *p* < .05; η ^2^_G_ = .27). Performance was significantly lower in the incongruent condition (*M* = .59; *SD* = .21) compared with the congruent condition (*M* = .71; *SD* = .17). The results of the post-hoc analyses for the rPT bins are reported Table 2. Additionally, the interaction between the congruency condition and the rPT bin was significant (*F*(5, 20) = 9.32 with *p* < .05 with Greenhouse-Geisser correction; η ^2^_G_ = .29). We calculated the congruency effect as the difference in performance between the congruent and incongruent condition in order to investigate the significant interaction effect from the previous ANOVA. The congruency effect was largest for rPTs from 200 to 300 ms, confirming results of the permutation test (see Fig 5A). However, pairwise comparisons of the congruency effect over the rPT bins did not reach significance, although the ANOVA revealed a significant interaction of the congruency and the rPT bin. This may be attributable to the lower statistical power in pairwise comparisons. The results of the post-hoc analyses are reported in Table 3.

**Table 2:** Post hoc tests for the main effect of the rPT bins in the Spatial Stroop task.

rPT bin	rPT bin	*t*	*df*	*p*	adj. *p*	adj. *p* signif.	*d*
50	150	0.50	9	.632	1.00	ns	0.16
50	250	-0.18	9	.858	1.00	ns	-0.06
50	350	-3.48	9	.007**	0.10	ns	-1.10
50	450	-9.19	9	<.001***	0.00	***	-2.91
50	550	-6.36	9	<.001***	0.00	**	-2.01
150	250	-0.35	9	.737	1.00	ns	-0.11
150	350	-3.64	9	.005**	0.08	ns	-1.15
150	450	-8.62	9	<.001***	0.00	***	-2.73
150	550	-6.62	9	<.001***	0.00	**	-2.09
250	350	-1.70	9	.124	1.00	ns	-0.54
250	450	-4.41	9	.002**	0.03	*	-1.39
250	550	-4.26	9	.002**	0.03	*	-1.35
350	450	-4.26	9	.002**	0.03	*	-1.35
350	550	-3.47	9	.007**	0.10	ns	-1.10
450	550	-1.15	9	.279	1.00	ns	-0.36

*Note.* Adjusted *p* values were calculated using Bonferroni correction.

* p < .05, ** p < .01, *** p < .001, **** p < .0001.

**Table 3:** Post hoc tests for the congruency effect over the rPT bins in the Spatial Stroop task.

rPT bin	rPT bin	*t*	*df*	*p*	adj. *p*	adj. *p* signif.	*d*
50	150	-6.07	4	.004**	0.06	ns	-2.71
50	250	-4.41	4	.012*	0.17	ns	-1.97
50	350	-5.88	4	.004**	0.06	ns	-2.63
50	450	-0.90	4	.419	1.00	ns	-0.40
50	550	-2.30	4	.083	1.00	ns	-1.03
150	250	-2.92	4	.043*	0.65	ns	-1.30
150	350	-0.84	4	.446	1.00	ns	-0.38
150	450	2.63	4	.058	0.87	ns	1.18
150	550	1.14	4	.319	1.00	ns	0.51
250	350	2.26	4	.087	1.00	ns	1.01
250	450	3.23	4	.032*	0.48	ns	1.44
250	550	2.57	4	.062	0.93	ns	1.15
350	450	4.74	4	.009**	0.14	ns	2.12
350	550	2.13	4	.100	1.00	ns	0.95
450	550	-2.52	4	.066	0.98	ns	-1.13

*Note.* Adjusted *p* values were calculated using Bonferroni correction.

* p < .05, ** p < .01, *** p < .001, **** p < .0001.

**Fig 3:**
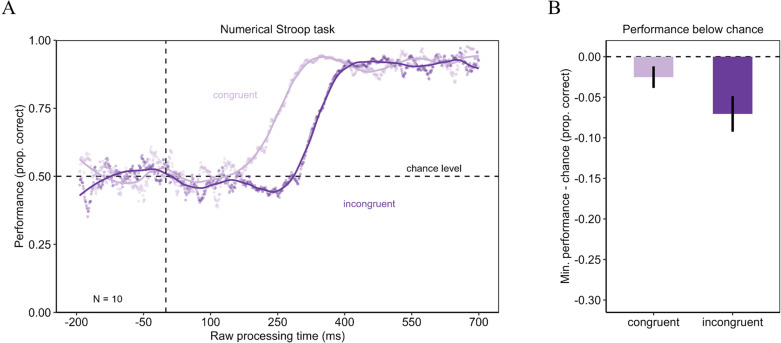
Urgency evokes dominance of stimulus-driven information in a Numerical Stroop task. Panel A shows the tachometric functions for the Numerical Stroop task in the congruent and incongruent condition of the Experiment 2. In the congruent condition performance started around chance level and then increased with increasing rPT. In the incongruent condition, however, the performance started at chance level but then dropped below chance level before the performance recovered and increased with increasing rPT. Panel B shows the difference of the minimum of the tachometric functions minus chance level for the congruent and the incongruent condition. The incongruent condition evoked a larger drop below chance level. Error bars are standard errors from a bootstrap.

### Experiment 2: Numerical Stroop task

In the Numerical Stroop task, the results from Experiment 1 reappeared (see Fig 3A). For both congruency conditions, the tachometric functions fluctuated around chance for negative and short rPTs. Beginning with a rPT around 150 ms, the tachometric functions progressed differently. In the congruent function, performance directly increased to near-perfect performance. In the incongruent condition performance dropped below chance around 250 ms rPT. After this drop, the tachometric function increased with increasing rPT to near-perfect performance. We compared the minima of the tachometric curves using a permutation test. Performance in the incongruent condition (minimum = .43) dropped significantly deeper below chance than performance in the congruent condition (minimum = .47) with *p* < .05 and *d*_*z*_ = 2.27 (see Fig 3B). In the Numerical Stroop task urgency opens-up a time-window, where responses are mainly driven by the irrelevant physical size of the stimuli instead of the task-relevant numerical size.

The repeated measures ANOVA was computed for rPTs from 0 ms to 700 ms. The performance was significantly higher in the congruent condition (*M* = .78; *SD* = .19) than in the incongruent condition (*M* = .68; *SD* = .24) with *F*(1, 9) = 15.48 and *p* < .05 (η ^2^_G_ = .19). Also, the main effect of the rPT bin (*F*(6, 54) = 113.29 with *p* < .05 with Greenhouse-Geisser correction; η ^2^_G_ = .73) as well as the interaction between the rPT bin and the congruency condition (*F*(6, 54) = 5.64 with *p* < .05 with Greenhouse-Geisser correction; η ^2^_G_ = .23) reached significance. The results of the post-hoc analyses for the rPT bins are reported in Table 4. As presented in Fig 5B when comparing the congruency effect over the rPT bins, the congruency effect was larger for rPTs from 200 to 300 ms than for nearly any other rPT bin, confirming results of the permutation test. The results of the post-hoc analyses are reported in Table 5.

**Table 4:** Post hoc tests for the main effect of the rPT bins in the Numerical Stroop task.

rPT bin	rPT bin	*t*	*df*	*p*	adj. *p*	adj. *p* signif.	*d*
50	150	0.57	19	.576	1.00	ns	0.13
50	250	-1.51	19	.147	1.00	ns	-0.34
50	350	-7.76	19	<.001***	0.00	****	-1.73
50	450	-11.28	19	<.001***	0.00	****	-2.52
50	550	-12.98	19	<.001***	0.00	****	-2.90
50	650	-12.45	19	<.001***	0.00	****	-2.78
150	250	-1.71	19	.104	1.00	ns	-0.38
150	350	-6.44	19	<.001***	0.00	****	-1.44
150	450	-8.35	19	<.001***	0.00	****	-1.87
150	550	-9.34	19	<.001***	0.00	****	-2.09
150	650	-8.29	19	<.001***	0.00	****	-1.85
250	350	-4.24	19	<.001***	0.01	**	-0.95
250	450	-6.65	19	<.001***	0.00	****	-1.49
250	550	-6.98	19	<.001***	0.00	****	-1.56
250	650	-5.96	19	<.001***	0.00	***	-1.33
350	450	-2.81	19	.011*	0.23	ns	-0.63
350	550	-4.02	19	.001***	0.01	*	-0.90
350	650	-3.05	19	.007**	0.14	ns	-0.68
450	550	-3.00	19	.007**	0.15	ns	-0.67
450	650	-0.93	19	.365	1.00	ns	-0.21
550	650	0.44	19	.666	1.00	ns	0.10

*Note.* Adjusted *p* values were calculated using Bonferroni correction.

* p < .05, ** p < .01, *** p < .001, **** p < .0001.

**Table 5:** Post hoc tests for the congruency effect over the rPT bins in the Numercial Stroop task.

rPT bin	rPT bin	*t*	*df*	*p*	adj. *p*	adj. *p* signif.	*d*
50	150	-1.40	9	.196	0.20	ns	-0.44
50	250	-3.00	9	.015*	0.01	*	-0.95
50	350	-1.37	9	.204	0.20	ns	-0.43
50	450	0.71	9	.497	0.50	ns	0.22
50	550	0.21	9	.836	0.84	ns	0.07
50	650	1.00	9	.345	0.34	ns	0.32
150	250	-1.84	9	.099	0.10	ns	-0.58
150	350	0.08	9	.941	0.94	ns	0.02
150	450	1.67	9	.130	0.13	ns	0.53
150	550	1.30	9	.225	0.22	ns	0.41
150	650	2.03	9	.073	0.07	ns	0.64
250	350	2.67	9	.025*	0.02	*	0.85
250	450	5.13	9	.001***	0.00	***	1.62
250	550	4.19	9	.002**	0.00	**	1.32
250	650	6.75	9	<.001***	0.00	****	2.13
350	450	6.15	9	<.001***	0.00	***	1.95
350	550	3.62	9	.006**	0.01	**	1.15
350	650	6.03	9	<.001***	0.00	***	1.91
450	550	-2.15	9	.060	0.06	ns	-0.68
450	650	0.63	9	.543	0.54	ns	0.20
550	650	1.25	9	.244	0.24	ns	0.39

*Note.* Adjusted *p* values were calculated using Bonferroni correction.

* p < .05, ** p < .01, *** p < .001, **** p < .0001.

**Fig 4:**
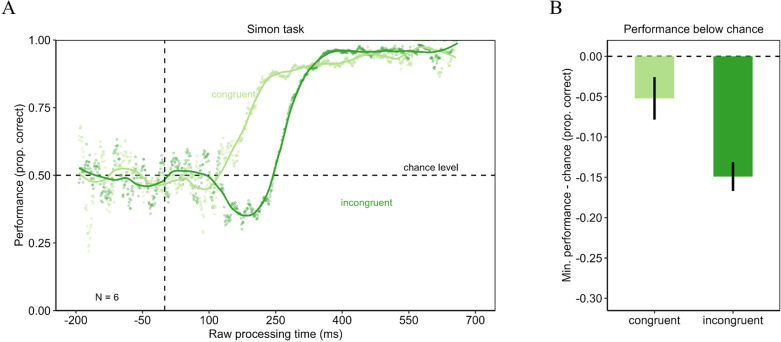
Urgency evokes dominance of stimulus-driven information in a Simon task. Left panel shows the tachometric functions for the Simon task in the congruent and incongruent condition. In the congruent condition performance started around chance level and then increased with increasing rPT. In the incongruent condition, however, the performance started at chance level but then dropped below chance level before the performance recovered and increased with increasing rPT. Right panel shows the difference of the minimum of the tachometric functions minus chance level for the congruent and the incongruent condition. The incongruent condition evoked a larger drop below chance level. Error bars are standard errors from a bootstrap.

### Experiment 3: Simon task

In Experiment 3, the results of Experiments 1 and 2 could be replicated. The tachometric functions are shown in Fig 4A. In both congruency conditions, performance fluctuated around chance level for negative and short rPTs. From around 150 ms rPT onwards, performance in the congruent condition increased. In the incongruent condition, performance dropped clearly below chance level. After that drop, performance recovered and progressed monotonically until it reached an asymptote close to perfect performance. The minima of the tachometric functions were compared using a permutation test. Performance dropped deeper below chance in the incongruent condition (minimum = .35) than in the congruent condition (minimum = .45, see Fig 4B). This difference was statistically significant (*p* < .001; *d*_*z*_ = 3.25). In high-urgency situation, reactions in the Simon task are driven by the position instead of the stimulus color, evoking a drop of performance below chance.

**Fig 5:**
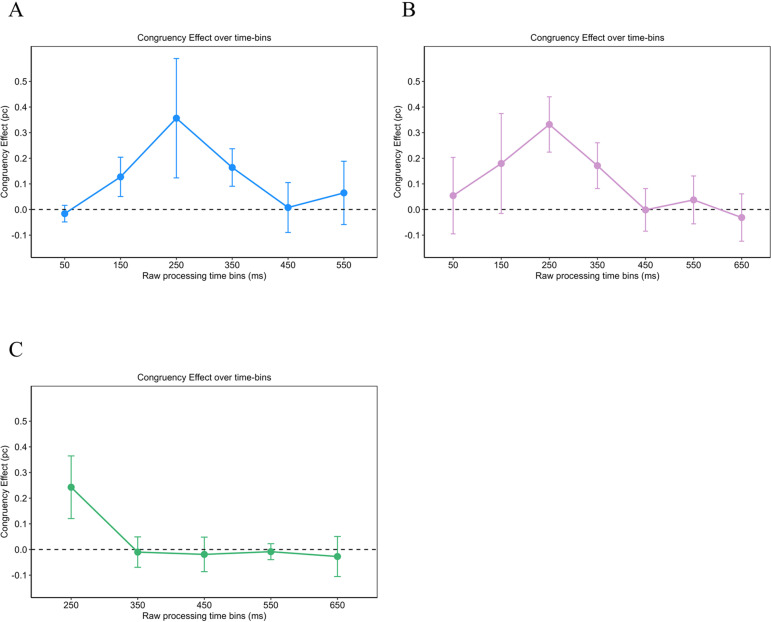
Congruency effects are larger for rPTs from 200 to 300 ms. Comparing the Congruency Effect, here defined as the difference in proportion of correct reactions between congruent and incongruent trials, over the rPT bins, showed a larger congruency effect for rPTs from 200 to 300 ms as for other rPT bins. This pattern was similar in all experiments. Panel A refers to the Spatial Stroop task with letter stimuli while Panel B represents the Numerical Stroop task. Results of the Simon task are shown in Panel C. Error bars indicate the 95% confidence interval for within-designs [42].

In Experiment 3, the repeated measures ANOVA was computed for rPTs from 200 ms to 700 ms. Unfortunately, data of some participants were missing for earlier rPTs, so that they could not be included in the analysis. Performance did not differ significantly between the congruent (*M* = .91; *SD* = .08) and the incongruent condition (*M* = .88; *SD* = .15) with *F*(1, 5) = 3.71 with *p* = .112 (η ^2^_G_ = .05). The ANOVA revealed a significant main effect of the rPT bin on performance (*F*(4, 20) = 11.98 with *p* < .05 with Greenhouse-Geisser correction; η ^2^_G_ = .42). The interaction between the congruency condition and the rPT bin was significant (*F*(4, 20) = 14.82 with *p* < .05 with Greenhouse-Geisser correction; η ^2^_G_ = .30). The results of the post-hoc analyses for the rPT bins are reported in Table 6. Results of the comparison of the congruency effect are shown in Fig 5C. The congruency effect was larger for rPTs from 200 to 300 ms compared with all other rPT bins. The results of the post-hoc analyses are reported in Table 7. This confirms the results of the permutation test, showing that performance dropped below chance in the incongruent condition under urgency.

**Table 6:** Post hoc tests for the main effect of the rPT bins in the Simon task.

rPT bin	rPT bin	*t*	*df*	*p*	adj. *p*	adj. *p* signif.	*d*
250	350	-2.55	11	.027*	0.27	ns	-0.74
250	450	-2.75	11	.019*	0.19	ns	-0.80
250	550	-3.27	11	.008**	0.07	ns	-0.94
250	650	-3.34	11	.007**	0.07	ns	-0.96
350	450	-2.09	11	.061	0.61	ns	-0.60
350	550	-5.00	11	<.001***	0.00	**	-1.44
350	650	-3.13	11	.010*	0.10	ns	-0.90
450	550	-1.23	11	.244	1.00	ns	-0.36
450	650	-2.54	11	.028*	0.28	ns	-0.73
550	650	-1.53	11	.155	1.00	ns	-0.44

*Note.* Adjusted *p* values were calculated using Bonferroni correction.

* p < .05, ** p < .01, *** p < .001, **** p < .0001.

**Table 7:** Post hoc tests for the congruency effect over the rPT bins in the Simon task.

rPT bin	rPT bin	*t*	*df*	*p*	adj. *p*	adj. *p* signif.	*d*
250	350	4.08	5	.010*	0.01	**	1.67
250	450	4.23	5	.008**	0.01	**	1.73
250	550	5.10	5	.004**	0.00	**	2.08
250	650	5.33	5	.003**	0.00	**	2.18
350	450	0.59	5	.581	0.58	ns	0.24
350	550	-0.11	5	.917	0.92	ns	-0.04
350	650	0.45	5	.674	0.67	ns	0.18
450	550	-0.60	5	.575	0.57	ns	-0.24
450	650	0.18	5	.862	0.86	ns	0.07
550	650	0.53	5	.621	0.62	ns	0.21

*Note.* Adjusted *p* values were calculated using Bonferroni correction.

* p < .05, ** p < .01, *** p < .001, **** p < .0001.

## Discussion

The present study aimed to investigate, whether urgency affects cognitive control also in tasks with stimuli other than arrows. Therefore, the urgency paradigm used by Salinas et al. [5] and Poth [6] was applied to a Spatial Stroop task with word stimuli in Experiment 1, a Numerical Stroop task in Experiment 2 and a Simon task in Experiment 3. In all experiments, urgency evoked a time-window, in which reactions were mainly stimulus-driven and therefore, in the incongruent condition, performance dropped below chance level. In all experiments, performance in the congruent condition was around chance for negative to short rPTs. Afterwards, performance increased with increasing rPT, reaching near-perfect performance. In the incongruent condition, performance fluctuated around chance for negative and short rPTs as well. Then performance dropped clearly below chance level in a certain time-window of around 250 ms rPT. After this drop, performance increased and, like in the congruent condition, reached near-perfect performance.

In the Spatial Stroop task with word stimuli, a conflict arises between the lexical meaning of the word and its spatial position. In the Numerical Stroop task, the cognitive conflict emerges between the numerical size of the presented numbers and their physical size. In the Simon task, the stimulus position conflicts the reaction to the stimulus color. Thus, the urgency-induced impairment of performance seems to emerge in a number of tasks and is not specific for arrow stimuli.

Compared to the arrow stimuli used previously, neither numbers nor colors are as highly associated with spatial directions as arrows. Arrows are overlearned symbols, which can automatically guide spatial attention [8,23,24]. At this point, it should be discussed that the words used in the Spatial Stroop task can be associated with spatial directions as well [8]. However, the numbers in the Numerical Stroop task are not that directly associated with spatial directions (especially since numbers and locations were counterbalanced, so that classic associations of lower and higher numbers with the left or right visual space [43,44] were controlled for). The same holds true for the colors in the Simon task. In all tasks, urgency likewise evoked a time-window in which the reaction to the stimuli were mainly stimulus-driven and goal-directed action was impaired, indicating that no spatial associations of the stimuli are required for the stimulus-driven information to dominate the response under urgency.

In addition, processing of words and numbers in the corresponding tasks differs from the processing of arrows. Word stimuli require several processing stages until the meaning of the word itself is understood. First, the single letters are perceived (including lower level stages of vision, figure-ground segregation, etc. [45,46]). In a second step, however, a semantic meaning must be assigned to the composition of the letters, which as a third step results in an integrated representation of the physical outlines and the lexico-semantic information finally resulting in the understanding of the word [47]. Similarly, the processing of numbers in the Numerical Stroop Task requires several processing steps. First, the numbers numerical and physical magnitude are recognized in parallel, then they are converted into an integrated representation in a second step. Finally the two presented numbers must be compared [27–29]. Our results show that even if the stimuli require different types of processing, urgency leads to a dominance of stimulus-driven information over goal-directed information and eventually to the impairment of goal-driven action.

Since we analyzed the performance over the rPT using both a tachometric analysis as well as a repeated measures ANOVA we would like to highlight the different interpretations that each analysis allows. The repeated measures ANOVA only shows that for a specific time window (around 200 to 300 ms rPT) there is a particularly large congruency effect (i.e. worse performance in the incongruent condition than in the congruent condition). Importantly, the tachometric function additionally shows that in this time window performance in the incongruent condition even drops below chance level. A significantly worse performance in the incongruent condition indicated that there is a strong cognitive conflict in the corresponding time window. In contrast, a performance below chance in the incongruent condition additionally means that stimulus-driven processing dominated the response.

The drop of performance below chance indicates that in this specific time interval, the reaction is mainly driven by the irrelevant stimulus information instead of the goal-driven information. This result must be differentiated from the classic speed-accuracy trade-off. The speed-accuracy trade-off describes the phenomenon that performance is better for slower reactions and decreases for faster reactions [48] and is quite general to multiple tasks [49]. Urgency evokes a similar pattern for congruent trials. For very fast reactions, performance in terms of proportion of correct reactions is low and then increases with increasing rPTs. However, in incongruent trials our results go beyond the speed-accuracy trade-off. Performance is around chance for very short rPTs. Then, with increasing rPT, performance first drops below chance - contrary to the predictions of the speed-accuracy trade-off. Only thereafter performance increases with increasing rPT. Accuracy under urgency is not only lower than for slow reactions, as it would be proposed by the speed-accuracy trade-off, but it is even below chance. This indicates that participants do not just make more errors but mainly execute the wrong reaction under urgency.

One aspect, however that the present study cannot answer, is the question to what extent stimulus-driven dominance in high-urgency situations can be transferred to tasks without any spatial component at all. In fact, given the strong dominance of stimulus-driven over goal-directed processing for visual stimuli capturing the gaze [5] and interactions with eye movement control [7], one can hypothesize that urgency specifically affected performance in spatial tasks. In the Spatial Stroop task and the Simon task the spatial dimension is the component that drives the conflict and thus dominates the response under high urgency. In the Numerical Stroop task, the spatial position is irrelevant to the conflict, but still ultimately determines which response must be executed by pressing the mouse button that matches the spatial position of the numerically larger number. Thus, it should be noted that in all tasks used here, the spatial location is an irrelevant dimension, that may cause the interference. Since the irrelevant dimension is largely responsible for inducing the cognitive conflict, it remains open whether urgency also affects cognitive control in tasks that are not spatially related.

Therefore, in future research, the irrelevant dimension should be varied in order to investigate if the effect of urgency on cognitive can be generalized to tasks that are based on different irrelevant dimensions. A recent study showed that spatial attention and the cognitive conflict in spatial cognitive control tasks rely on similar systems [7]. The performance in a Spatial Stroop task was compared between blocks of trials with an additional fixation task on top and blocks where the eyes could be moved freely. The results showed that the congruency effect, in this case defined as the difference in reaction times between congruent and incongruent trials, was reduced in blocks with fixation task. The results were interpreted as a reduction of processing of the irrelevant stimulus location thus reducing the conflict. This could be attributed to the fact that holding fixation ties up attentional resources at the fixation location, leaving fewer resources available for processing the stimulus location. This attentional disinhibition effect shows that the conflict-driving component, i.e., the location, relies on spatial attention systems. Thus, no conclusion can be drawn about complete generalizability of the effect of urgency on all cognitive control tasks. Since this question remains open for further research, in future studies the urgency paradigm should be applied to further cognitive control tasks like the classic color-word Stroop task.

## Conclusion

These results reveal that the effect of urgency on cognitive control occurs also in tasks that do not use arrow stimuli. Accordingly, urgency evokes a dominance of stimulus-driven action over goal-directed action across a number of spatially related cognitive control tasks with different stimuli. As such, the results suggest that urgency affects cognitive control quite generally.

## Supporting information

S1 Data Individual results of experiment 2A and 2B. (PDF)
